# O2-8 Effects of a 12-week walking football intervention on cardiovascular disease risk factors in an older adult population: a randomised controlled trial in the UK

**DOI:** 10.1093/eurpub/ckac094.016

**Published:** 2022-08-29

**Authors:** Tom McBain, David Broom

**Affiliations:** Department of Sport and Physical Activity, Sheffield Hallam University, Sheffield, United Kingdom

**Keywords:** walking football, cardiometabolic disease, community setting, controlled trial

## Abstract

**Background:**

Interventions promoting physical activity and improved wellbeing delivered through professional football clubs have previously produced favourable changes in health in adult males (Hunt et al., 2014). Walking football has proven a popular variant suitable for older adults and studies have reported positive changes in health outcomes (Arnold et al., 2015; Reddy et al., 2017; McEwan et al., 2019). However, studies exploring outcomes following walking football interventions when compared to a control group in an older adult population are scarce. Therefore, the aim of the current study was to explore the effects of a 12-week walking football intervention on cardiovascular disease risk factors and quality of life in an older adult population.

**Methods:**

Following ethical approval, participants (n = 23, one female; 68 ± 8 years old), were recruited through the Drink Wise, Age Well scheme; a local Lottery funded organisation and were invited to take part in a 12-week walking football intervention or control group. Intervention group participants (n = 17), were invited to one 60-minute walking football session per week consisting of a warm-up and small-scale competitive games, whereas control group participants (n = 6), continued with their normal habitual lifestyle. Outcomes measured were weight, resting blood pressure, non-fasting blood lipids, six-minute walk distance, and quality of life. Outcomes were assessed at baseline (week-0), midpoint (week-7) and post-intervention (week-13). Sessional RPE was recorded after each session. Preliminary data were analysed using a repeated-measures ANOVA.

**Results:**

Adherence to the 12-week intervention was 83%. Mean (± *SD*) attendance for sessions was 68 ± 26% (range; 0-92%). Mean rating of perceived exertion was 6.3 ± 1 (range; 2-8). Analysis revealed a significant reduction in total cholesterol (p = 0.01) and LDL cholesterol (p = 0.19) in the intervention group. However, despite favourable changes in both groups for each variable, no significant difference between groups occurred between baseline, week-7 and post-intervention.

**Conclusion:**

The study outlines the potential for individuals enrolled in a walking football scheme to engage in weekly moderate-to-vigorous PA as well as maintaining and improving a myriad of health outcomes. Engagement in other pre-existing health promotion schemes, such as walking football at other local organisations may have influenced outcome measures.

